# Pharmacological and psychological approaches to insomnia treatment in cardiac patients: a narrative literature review

**DOI:** 10.3389/fpsyt.2025.1490585

**Published:** 2025-02-13

**Authors:** Britta Stapel, Marlies E. Alvarenga, Kai G. Kahl

**Affiliations:** ^1^ Department of Psychiatry, Social Psychiatry and Psychotherapy, Hannover Medical School, Hannover, Germany; ^2^ Institute of Health and Wellbeing, Federation University Australia and Victorian Heart Institute, Melbourne, VIC, Australia

**Keywords:** insomnia, cardiovascular disease, pharmacological treatment, benzodiazepine, orexin receptor antagonist, antidepressant, Z-drug, melatonin

## Abstract

Sleep disorders are highly prevalent in the general population and are considered a major public health issue. Insomnia constitutes the most frequent sleep disorder in healthy individuals and has been shown to be even more frequent in patients with physical illnesses including cardiovascular diseases. Inadequate sleep quality and short sleep duration, independent of underlying causes, have been linked to the development and progression of cardiometabolic disorders. Additionally, insomnia has been found to be associated with adverse outcome measures, including daytime sleepiness, fatigue, decreased self-reported physical functioning, lower exercise capacity, poor health related quality of life, depressive symptoms, higher rates of hospitalization and increased mortality in patients with cardiovascular diseases. Against this background, comparatively little information is available in the literature regarding the treatment of chronic insomnia in cardiac patient populations. While guidelines for the general population suggest cognitive behavioral therapy for insomnia as a first-line treatment option and preliminary evidence suggests this treatment to be beneficial in cardiac patients with insomnia symptoms, it is often limited by availability and possibly the clinician’s poor understanding of sleep issues in cardiac patients. Therefore, pharmacologic treatment remains an important option indicated by the high number of hypnotic drug prescriptions in the general population and in patients with cardiovascular disorders. In this narrative review of the literature, we summarize treatment options for chronic insomnia based on clinical guidelines for the general population and highlight necessary considerations for the treatment of patients with cardiovascular diseases.

## 1 Introduction

### 1.1 Chronic insomnia disorder

In the past decades, sleep and its impact on physical and mental health has increasingly become a research focus. It is now consensus that sleep disturbances negatively impact the quality of life, mood, and cognitive function and are associated with a heightened risk for somatic diseases (1). An expert panel convened by the United States-based National Sleep Foundation has suggested an appropriate sleep duration of 7-9 h/night for adults between the ages of 25 and 65 years (2). Contrarily, data indicate that sleep duration in the general population is comparably shorter, with 35% of adults reporting to sleep less than 6 h/night, and an additional 30% indicating a sleep duration of just 7 h/night (3). On the one hand, lifestyle habits associated with modern day society, including work schedules, unhealthy diet, smoking, lack of physical exercise, increasing use of electronic devices, and excessive psychosocial stress, are known to negatively impact sleep duration in adults (4). On the other hand, sleep disorders are highly prevalent in the general population and are therefore considered a major public health issue (5). Insomnia constitutes the most frequent sleep disorder (6). Based on the revised 5^th^ edition of the Diagnostic Statistical Manual of Mental Disorders (DSM-5-TR) and the 3^rd^ edition of the International Classification of Sleep Disorders (ICSD-3) diagnosis of insomnia requires the presence of at least one of the following criteria: difficulty initiation sleep, maintaining sleep, or early awakening. Additionally, at least one associated impairment in daytime functioning, for example a reduction in cognitive performance, fatigue, or disturbances in mood, have to be present (7, 8). The diagnosis of chronic insomnia requires the presence of dissatisfying sleep quality or sleep quantity for at least three nights a week over a time period of at least three months when adequate opportunity for sleep is given (7, 8). Finally, with the introduction of DSM-5 and based on a National Health Institute (NIH) conference on insomnia in 2005, the previous distinction between primary and secondary insomnia (referring to insomnia related to another somatic or mental disorder) was removed (9). Acute insomnia as a reaction to acute stressors is common, affecting 30-50% of the population in industrialized countries, and mostly resolves after cessation of the stressor (10). While acute insomnia does not require specific treatment, chronic insomnia necessitates clinical attention regardless of potential underlying causes.

The prevalence of chronic insomnia in the general population is estimated to be at least 5-10% in industrialized countries (11, 12), it has been found to be twice as common in women compared to men, and prevalence has been shown to increase with age (13). Compared to the general population, higher prevalence rates have been reported in the context of psychiatric and somatic illnesses. In this regard, data from general practice in Germany and Norway showed a prevalence of 20% and 50%, respectively (14, 15).

Chronic insomnia places a significant financial burden on healthcare systems in developed countries. It is associated with a marked impairment in functional status (16, 17), with increased absences from work (18), and with heightened occurrence of workplace and motor vehicle accidents (19, 20). Additionally, insomnia significantly increases the risk for psychiatric disorders (21, 22). According to data from the World Health Organization (WHO), insomnia ranked 11^th^ on the list of brain disorders in terms of global burden and 9^th^ among neuropsychiatric disorders in terms of disability-adjusted life years (DALYs) (23). In Europe, sleep disorders ranked 9^th^ among brain disorders in 2010 regarding direct and indirect costs (24).

### 1.2 Insomnia and cardiometabolic disease

Inadequate sleep and sleep deprivation, independent of underlying causes, have been linked to the development and progression of cardiometabolic disorders. Several meta-analyses have confirmed that insomnia is associated with an increased cardiovascular disease risk (25–27). In particular, insomnia has been found to be a risk factor for arterial hypertension, myocardial infarction, and heart failure (28–30). Furthermore, insomnia has been associated with an increased risk for type 2 diabetes (31). Next to insomnia, short sleep duration defined as less than 6 h of sleep per night has been indicated as a risk factor for cardiometabolic conditions and health behaviors that predispose to cardiovascular disease, including increased calorie intake, obesity, type 2 diabetes, and hypertension (32–37). Finally, short sleep duration was found to increase the risk for cardiovascular disease (36). Based on the mounting evidence that indicates a significant connection between sleep and cardiovascular disease, sleep disturbances have been proposed as the 10^th^ modifiable cardiovascular risk factor (38).

**Figure 1:**
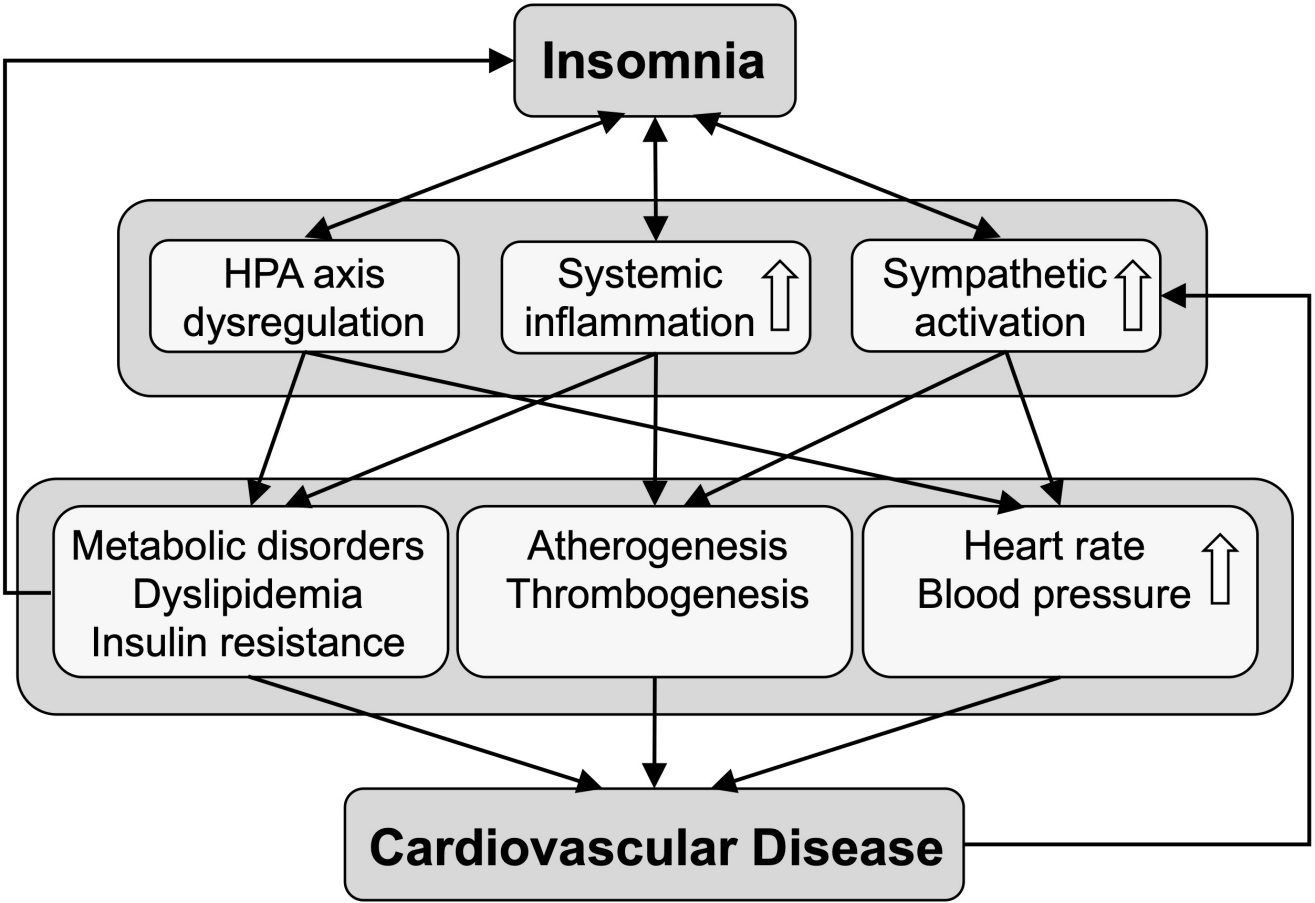
The scheme depicts biological pathways linking insomnia to cardiovascular disease. Insomnia is bidirectionally associated with dysregulation of the hypothalamus-pituitary-adrenal (HPA) axis, and with increased systemic inflammation and sympathetic activation, leading to various cardiometabolic conditions that predispose to cardiovascular disease.

Similar to other mental disorders, including major depressive disorder and symptoms of depression, the association between insomnia and cardiometabolic diseases appears to be bidirectional, i.e. while individuals that suffer from insomnia and insomnia symptoms are more likely to develop cardiometabolic disorders, cardiac patient populations also have been shown to present with insomnia at higher prevalence rates than observed in the general population (39–41). Mechanisms and biological pathways involved in the bidirectional relationship have been extensively reviewed before (42, 43) and are summarized in **Figure 1**.

In this regard, it is estimated that nearly 45% of patients that are diagnosed with cardiovascular disease have insomnia (44). Importantly, the presence of sleep disturbances and insomnia has been found to be associated with adverse outcome measures, including higher rates of hospitalization and increased mortality in patients with coronary heart disease (45, 46).

In the context of cardiovascular diseases, the impact of insomnia and insomnia symptoms on outcome measures has been most extensively studied in patients with heart failure. In this regard, a current integrative review suggests that insomnia and insomnia symptoms are associated with daytime sleepiness, fatigue, decreased self-reported physical functioning, lower exercise capacity, poor health related quality of life, depressive symptoms, and cardiac events (40).

Taking into account the high prevalence of insomnia and the associated adverse outcome measures, comparatively little information is available in the literature regarding treatment of chronic insomnia in cardiac patient populations.

#### 1.2.1 Sleep disordered breathing and cardiovascular medication

Importantly, next to insomnia, sleep-related breathing disorders are common in patients with cardiovascular diseases and can be associated with sleep disruption due to repeated arousals during the night (47). Obstructive sleep apnea (OSA) constitutes the most frequent form of sleep disordered breathing in patients with cardiovascular disorders and occurs in over 40% of patients (48). It is caused by repetitive closure of the upper airways during sleep. On the one hand this results in episodes of obstructive apneas, hypoapneas, or respiratory effort-related arousal. On the other hand, OSA constitutes the main cause of chronic intermittent hypoxia, which has been linked to the pathology of several cardiovascular conditions, including atherosclerosis, endothelial dysfunction, hypertension, insulin resistance, dyslipidemia, carotid intima-media thickness, and inflammation (49). Underlying mechanisms and pathways are reviewed in a recent dedicated publication by Assallum and colleagues (49). Accordingly, OSA has been found to be associated with heightened mortality and overall cardiovascular disease risk (50). Furthermore, an increased risk for hypertension, arrhythmias, coronary events and adverse outcome measures following myocardial infarction have been reported (50). Therefore, particularly in individuals that present with cardiovascular disease, diagnosis of sleep disorder breathing and OSA need to be considered, when excessive daytime sleepiness, fatigue, or snoring are present. Treatment options, including weight loss and continuous positive airway pressure (CPAP) therapy, have been found to likely improve parameters of quality of life and daytime sleepiness in patients with OSA and may benefit control of hypertension and paroxysmal atrial fibrillation. Contrarily, in the absence of daytime sleepiness or nocturnal hypoxemia studies failed to demonstrate positive effects of OSA treatment on cardiovascular endpoints (50).

Position papers and recommendations on the treatment of sleep disorders in cardiac patients mainly focus on the diagnosis and treatment of sleep-related breathing disorders (51, 52), owing to the well-established association of sleep-disordered breathing and cardiovascular morbidity (53). However, a study by Redeker and colleagues that reported an association of insomnia symptoms and quality of life parameters in patients with stable heart failure, concluded that these relationships could not be explained by sleep-disordered breathing (54). Additionally, the study highlights that in patients receiving guideline-based therapies for heart failure, insomnia symptoms often persist, and that therefore therapeutic management of heart failure alone is insufficient to diminish insomnia symptomology (54). Accordingly, current data suggest that between 10% and 20% of heart failure patients take hypnotic medications at least three times per week (55–57).

Next to underlying somatic causes of sleep disturbances, a potential association of cardiovascular drugs and insomnia symptoms might be considered. While there is comparatively little literature available, it has been shown that beta-blockers, which constitute indispensable drugs in the treatment of patients with cardiovascular disease, can be attributed to psychiatric adverse events. In fact, early studies found a decrease in nighttime melatonin release in healthy volunteers in response to beta-blocker administration, caused by direct inhibition of the beta1-adrenoreceptor (58). Accordingly, a recent meta-analysis found that beta-blocker treatment could possibly be associated with an increased risk for sleep disturbances and sleep disorders, including insomnia (59).

Additionally, diuretics could be discussed as drugs that may potentially increase the likelihood of insomnia via nocturia. The cause of nocturia is often multifactorial, and includes age-related changes in the genitourinary system (such as benign prostate hypertrophy), uncontrolled diabetes mellitus, vasopressin deficiency, fluid redistribution associated with edematous states such as congestive heart failure, chronic kidney disease, as well as sleep disorders itself. In a prospective study by Endeshaw and colleagues, nocturia due to several reasons was examined concerning sleep problems and mortality. In total, 1,478 patients aged 73.8 ± 2.9 years were included and followed prospectively over 10 years in average (60). During the follow-up period, 760 deaths occurred. The results of the study indicate that the association between three or more nycturia episodes and mortality is independent of insomnia symptoms and can be better explained by prevalent diabetes mellitus and pre-existing cardiovascular morbidity. Although it cannot be ruled out that use of diuretics could lead to sleep problems via nycturia, it seems unlikely that this may influence long-term prognosis of cardiovascular diseases (60). Furthermore, diuretics are commonly given during the day, to prevent/minimize the risk of nocturia.

Of note, a recent qualitative descriptive study that assessed self-reported factors influencing the sleep situation and management of sleep problems of 20 patients (mean age 73 years) with cardiovascular disease and insomnia found four underlying categories of sleep disruptors, i.e. cognitive, social, physical, and behavioral. While direct side effects of cardiovascular drugs were not specifically named, the wondering whether drug-related adverse effects might cause sleep problems was found as one cognitive event that triggered sleep disruptions. Furthermore, while nocturia was cited as a trigger for the behavioral category, this was attributed to fluid intake in the late afternoon or evening rather than to drug side-effects (61).

Against this background, recommendations regarding the treatment of chronic insomnia in patients with cardiovascular disorders, especially in those with heart failure, are missing.

## 2 Methods

This narrative review was conducted to explore the pharmacologic and psychologic approaches for insomnia treatment in cardiac patients. A systematic search of the literature was performed across three major databases: PubMed, Google Scholar, and the Cochrane Database of Systematic Reviews. Articles published up to July 31, 2023, were included.

The search strategy employed a combination of Medical Subject Headings (MeSH) terms and free-text keywords tailored to each database. **Supplementary Table 1** provides an overview regarding the keywords utilized for each of the three relevant elements, i.e. insomnia, cardiovascular disease, and treatment approach, which were used either singly or in combination. Titles and abstracts of identified articles were screened and full-text articles were retrieved for potentially eligible studies. To refine the search and to ensure comprehensive coverage of the topic, Boolean operators (AND, OR) were used.

The present narrative review is limited to articles written in English and it does not encompass a systemic or quantitative meta-analysis. Rather, this article aims to provide a broad and integrative perspective on current evidence.

## 3 Treatment options for insomnia

### 3.1 Psychological treatment of insomnia

Insomnia is a key symptom of psychological distress. Fernandez-Mendoza and Vgontzas proposed a connection between insomnia and the activation of stress responses (62). This link is particularly evident in individuals experiencing chronic hyperarousal and depression, leading to persistent difficulties with sleeplessness (63, 64). Clinical practice guidelines in the United States, Canada, and Europe recommend non-drug interventions, in particular cognitive behavioral therapy for insomnia (CBT-I), as the first-line treatment of chronic insomnia in adults of any age (65, 66). CBT-I can be applied either as face-to-face or as e-mental health intervention.

CBT-I is a relatively short-term (6-8 sessions), structured evidence-based, multi-component treatment approach to insomnia (67). CBT-I typically involves several techniques to target perpetuating factors and bring about a change in the behaviors and thoughts contributing to insomnia (68). These techniques include psychoeducation, stimulus control, sleep restriction, cognitive restructuring, and relaxation training (69). Stimulus control and sleep restriction are administered as synergistic therapeutic approaches. Stimulus control primarily focuses on effectively managing episodes of nocturnal wakefulness through behavioral modifications. On the other hand, sleep restriction aims to enhance the inclination to initiate sleep while facilitating the establishment of uninterrupted and consolidated periods of sleep (68).

The efficacy of CBT-I as a first-line treatment is well established in clinical guidelines (70). Numerous studies have demonstrated the efficacy of CBT-I in improving sleep outcomes. A recent meta-analysis by Feng and colleagues found that CBT-I demonstrated superiority over no treatment in addressing insomnia, although its relative efficacy compared to no treatment for depression remained uncertain (71). Moreover, the effectiveness of CBT-I appeared to be on par with hypnotics for managing both insomnia and depression (71). Notably, the authors highlighted that the noninvasive nature of CBT-I suggested the intervention is likely safe. However, the trials exhibited variability in methodological quality. Older adults often report trouble sleeping. Vgontzas and colleagues found that middle-aged men showed higher sensitivity to the arousing effects of corticotropin-releasing hormone (CRH) than younger men, which may explain physiologically the increased prevalence of insomnia in older subjects (72). A systematic review and meta-analysis examining the efficacy of CBT-I in older adults found that CBT-I was safe and effective in improving sleep outcomes for this population (73). This is an important consideration given that cardiovascular diseases tend to affect mostly people over 65 years of age (74). Offering CBT-I instead of prescribing medication for individuals experiencing insomnia has several advantages. While sleep medications can provide effective short-term relief for insomnia, certain patients encounter adverse effects such as amnestic episodes, cognitive decline, and residual drowsiness upon awakening (75). Moreover, a subset of individuals continues to grapple with sleep disturbances despite medication usage, often necessitating escalating doses and ultimately leading to dependency, overuse from increased tolerance and accidents caused by taking these drugs (76). In addition to improving insomnia symptoms, CBT-I has also been found to be effective in reducing depression, anxiety, chronic pain, and enhancing sleep-related quality of life (77–79).

CBT-I has also been found to be effective in treating insomnia in various populations, including individuals with chronic pain, comorbid psychiatric disorders such as depression and anxiety, and those with insomnia related to shift work or jet lag (80). This is important in the context of heart disease, as around one in five cardiac patients suffer from depression and anxiety (81) and shift workers tend to experience higher rates of heart disease and depression (82). Social determinants of health (i.e. housing, employment, social isolation) are also linked to sleep quality (83). Heenan and colleagues found that cardiac patients undergoing a CBT-I program reported improved sleep and significantly lower levels of anxiety and depression, indicating that this intervention is successful both for insomnia and overall emotional wellbeing (84). In addition, CBT-I has been found to be effective when delivered via telemedicine, making it more accessible to those who may have difficulty accessing in-person treatment (85). However, the findings of Kallestad et al. yielded inconclusive results in terms of the efficacy comparison between face-to-face delivery and telehealth-based administration of CBT-I (86).

According to Thomas et al. there is limited access to CBT-I providers (87). The authors estimated that in 2016 there were under one thousand CBT-I specialists worldwide, with the majority unequally distributed in the USA (87). Adequate training in CBT-I is important as although CBT-I is a relatively brief form of therapy based on principles of CBT, competence with the delivery of CBT-I in a cardiac population would be associated with better clinical outcomes (88).

While guidelines for the treatment of insomnia explicitly recommend CBT-I as the first-line treatment option for adults with insomnia, other psychotherapeutic approaches have been assessed for insomnia treatment. In this regard, mindfulness-based interventions (MBIs) have been suggested in insomnia treatment. Although mindfulness is most often defined as paying attention, on purpose, in the present moment without judgement, there appears to be little consensus on what constitutes mindfulness and what mechanisms underlie its effectiveness or how the construct is measured. Accordingly, a meta-analysis of randomized controlled trials that investigated the effect of MBIs on sleep among adults with insomnia or sleep problems found considerable conceptual and methodological heterogeneity among 13 included studies, which included diverse MBIs (i.e. mindfulness stress reduction, mindful bridging, mindfulness based cognitive behavioral therapy, mindfulness based therapy for insomnia, and acceptance and commitment therapy) in diverse patient populations with insomnia (89). Additionally, none of the included trials was designed to compare effects of MBIs to another active treatment of insomnia. However, MBIs appeared to be significantly more effective in improving insomnia symptoms compared to attention/education and waitlist controls (89). Another meta-analysis that assessed the effect of MBIs as a component of the CBT-I therapeutic system and that included randomized controlled trials as well as non-randomized studies, identified nine studies that were included in subsequent analyses. The meta-analysis revealed small to medium effect sizes on ISI scores that failed to reach significance and although several studies combined MBIs with CBT-I components, no positive effect on chronic insomnia was found compared to the respective control conditions (90).

Acceptance and Commitment Therapy (ACT) constitutes another form of third wave behavioral therapy discussed as a treatment option for insomnia, that however is also not currently recommended in the guidelines. ACT is based on a comprehensive scientific philosophy called functional contextualism, and includes six treatment processes, i.e. acceptance, defusion, self, now, values, and action, with the overall goal to improve psychological flexibility (91). A limited number of studies have assessed the effectiveness of ACT in the context of insomnia and insomnia symptoms. A recent systematic review that assessed the efficacy of ACT for insomnia treatment identified 11 studies of which seven combined ACT with behavioral and/or cognitive elements, while four applied ACT only. The authors conclude that ACT as monotherapy constitutes a ‘*possibly efficacious*’ treatment for insomnia, while the combination of ACT with behavioral components, including sleep restriction and stimulus control, constitutes a ‘*probably efficacious*’ treatment for insomnia (92).

While Paulos-Guarnieri and colleagues included randomized and non-randomized trials as well as case series and case reports, a meta-analysis by Ruan et al. limited included studies to randomized trials and identified 18 studies (93). The authors found that ACT was better for insomnia relieve compared to waitlist control, while no significant improvement of subjective sleep parameter were found. Furthermore, ACT was more beneficial than psychoeducation in improving insomnia and had similar effects compared to CBT. The effects of ACT and CBT on subjective sleep parameters did also not significantly differ. In patients with other illnesses, the effect of ACT on sleep quality was similar to findings in insomnia patients. However, while ACT and CBT had comparable impact on sleep quality at early follow-ups, after 12 months ACT was found to be less effective than CBT (93).

Overall, recent systematic reviews and meta-analyses suggest that ACT might be beneficial in the short-term treatment of insomnia, while its long-term effectiveness remains uncertain. Additionally, ACT does not appear to be more effective than CBT in insomnia treatment and particularly studies comparing ACT to CBT-I, which is considered the state-of-the-art intervention concerning psychotherapeutic treatment of insomnia are currently limited. In this regard, a recent randomized control trial compared an ACT-based protocol to waitlist controls as well as to CBT-I treatment (94). The authors describe a beneficial effect of ACT compared to waitlist controls. However, CBT-I remained superior to ACT regarding ISI scores at post-treatment as well as at six months follow-up. Therefore, ACT might constitute an effective treatment option for insomnia patients, particularly in those, who struggle to adhere to behavioral techniques such as sleep restriction and stimulus control (94).

### 3.2 Pharmacological treatment of insomnia

Short-term pharmacologic treatment of chronic insomnia, defined as a treatment duration of 4 to 5 weeks or less, might be considered if CBT-I is not applicable due to cost restrains or unavailability, reservations or inability of the patient to participate in the therapy, or in the case of non-response (65, 66, 95). Albeit the favorable benefit-to-risk ratio of CBT-I, pharmacologic treatment remains the most common approach to therapy (95). In this regard, an international survey found that a drug prescription was given to approximately 50% of patients in the US and Western Europe and to up to 90% of patients in Japan that consulted a physician regarding their sleep complaints (96). Accordingly, prescriptions of hypnotic medication and use of over-the-counter agents for treatment of insomnia have seen significant increases over the past two decades (5). Indeed, it is estimated that between 1999 and 2010 the number of prescriptions for any sleep medication increased by approximately 290% (97), thereby outpacing the increase in sleeplessness complaints and insomnia diagnoses (5). Finally, while a recent study from the US suggests a decline in the use of medications for sleep disturbances in recent years, especially in Food and Drug Administration (FDA)-approved drugs and particularly in older individuals aged 80+ years, pharmacologic treatment appears to remain the most frequent treatment approach for insomnia (98).

Against this background, a new, evidence-based Clinical Practice Guideline for the Pharmacologic Treatment of Chronic Insomnia in Adults (hereinafter referred to as *Clinical Practice Guideline*) was published in 2017 by the American Academy of Sleep Medicine (95). The guidelines are based on the GRADE methodology (Grading of Recommendations, Assessment, Development, and Evaluation) that takes into account quality of evidence, benefit versus harm of a given treatment, and patient values and preferences (99, 100), and represent a comprehensive and systematic analysis of single agents used for the treatment of chronic insomnia.

A variety of drugs are available for insomnia treatment. Those include prescription hypnotic drugs indicated for the treatment of insomnia, including benzodiazepine receptor agonists (BZRAs), chronobiotic drugs, and low-dose doxepin, as wells as prescription drugs with off-label usage for the treatment of insomnia, including antidepressants, antipsychotics, and anticonvulsants. Additionally, over-the-counter medications, including melatonin and antihistamines are commonly used (13).

The following section will discuss prescription medications approved by the FDA for the treatment of insomnia, as well as off-label treatments and over-the-counter options, and the according recommendations given in the Clinical Practice Guideline will be highlighted (95). Of note, all studies included in the guideline assessed the efficacy and/or safety of the respective medications as a short-term treatment (i.e. from one day to five weeks). The authors suggest that long-term use of newer generation BZRAs should be limited to individuals that have no access to or did not benefit from CBT-I treatment. For these cases a screening for potential contraindications has been performed and regular follow-ups are required (95). Finally, it has to be considered that the Clinical Practice Guideline is based on trials that assessed the respective medications for the treatment of primary chronic insomnia and did not include studies with patients that had significant comorbidities (95).

#### 3.2.1 Benzodiazepine receptor agonists

Benzodiazepine receptor agonists (BZRAs) constitute the most commonly prescribed, FDA-approved drugs for the treatment of insomnia and are among the most prescribed medications amongst older adults (101). BZRAs are hypnotic drugs that comprise benzodiazepines (BZs), characterized by a defining fused benzene and diazepine ring, and so-called Z-drugs that were introduced later and lack the classical BDZ structure. All BZRAs target the benzodiazepine-γ-aminobutyric acid (GABA)_A_ receptor and elicit their hypnotic effect by enhancing the sleep-promoting action of GABA (102).

##### 3.2.1.1 Benzodiazepines

BZs are allosteric modulations of (GABA)_A_ receptors. They act via the benzodiazepine side of the receptor (BZR) and are characterized by their sedative-hypnotic properties (103). The first classical BZs were introduced in the late 1950s and rapidly replaced barbiturates and related drugs as a safer option for anxiolytic and hypnotic treatment (104, 105). In the late 1970s, BZs constituted the most prescribed medications worldwide for treatment of anxiety, agitation, seizures muscle spasm, anesthesia premedication, and insomnia (106). Subsequently, with the more widespread use, reports regarding potential adverse effect of BZs, including addiction and misuse, became more frequent, which led to a significant decrease in prescriptions (107). However, recent research suggests that BZs constitute important and safe treatment options when prescribed to patients without substance use disorders and for appropriate indications (107, 108). More recent data from the US showed that BZs with FDA approval for insomnia treatment ranked third amongst insomnia prescriptions in 2009/2010. In this study, 0.4% of the sample population reported to have used a BZ at least once in the past month (101).

Six drugs belonging to the class of BZs were included in the Clinical Practice Guideline (95). All included BZs are FDA-approved, with indication for treatment of insomnia. Only temazepam was recommended for the treatment of sleep onset insomnia as well as sleep maintenance insomnia in adults (**Table 1**). Additionally, triazolam was recommended for the treatment of sleep onset insomnia. No recommendations were made for estazolam, flurazepam, oxazepam, and quazepam due to insufficient evidence regarding efficacy and/or safety (95).

**Table 1:** Drugs recommended for treatment of chronic insomnia disorder.

Treatment	Dose	Recommendation	Strength	Ref.
Benzodiazepines				
Temazepam (FDA-approved, off-label use for insomnia)	15 mg	Use is recommended for **sleep onset insomnia** and **sleep maintenance insomnia** (versus no treatment) in adults	WEAK	(109–111)
Triazolam (FDA-approved for treatment of insomnia)	0.25 mg	Use is recommended for **sleep onset insomnia** (versus no treatment) in adults	WEAK	(112)
Z-drugs				
Eszopiclone (FDA-approved for treatment of insomnia)	2 mg and 3 mg	Use is recommended for **sleep onset insomnia** and **sleep maintenance insomnia** (versus no treatment) in adults	WEAK	(113–118)
Zaleplon (FDA-approved for treatment of insomnia)	10 mg	Use is recommended for **sleep onset insomnia** (versus no treatment) in adults	WEAK	(119, 120)
Zolpidem (FDA-approved for treatment of insomnia)	10 mg*	Use is recommended for **sleep onset insomnia** and **sleep maintenance insomnia** (versus no treatment) in adults	WEAK	(114, 115, 117, 121–130)
Melatonin agonists				
Ramelteon (FDA-approved for treatment of insomnia)	8 mg	Use is recommended for **sleep onset insomnia** (versus no treatment) in adults	WEAK	(131–134)
Antidepressants (low-dose)				
Doxepin (FDA-approved for treatment of insomnia)	3 mg and 6 mg	Use is recommended for **sleep maintenance insomnia** (versus no treatment) in adults	WEAK	(135–139)
Orexin receptor antagonists				
Suvorexant (FDA-approved for treatment of insomnia)	10 mg 15/20 mg and 20 mg	Use is recommended for **sleep maintenance insomnia** (versus no treatment) in adults	WEAK	(140, 141)

The table depicts dose, recommendation and its respective strength, and studies with sufficient quality for drugs recommended for sleep onset insomnia and/or sleep maintenance insomnia in the Clinical Practice Guideline for the Pharmacologic Treatment of Chronic Insomnia in Adults (95). FDA, Food and Drug Association.

*Since the initial approval, recommended starting dose has been lowered to 5 mg for immediate-release zolpidem products, and recommended starting does for extended-release forms has been lowered from 12.5 mg to 6.25 mg.Bold text indicates whether recommendations relate to sleep maintenance or sleep onset insomnia, respectively.

##### 3.2.1.2 Z-drugs

Z-drugs owe their name to the fact that two of the three FDA-approved drugs of the class, i.e. zolpidem and zaleplon, begin with the letter Z, while eszopiclone constitutes the active stereoisomer of zopiclone (142). Z-drugs, while chemically heterogenous, constitute non-benzodiazepine BZRAs that were developed to improve pharmacokinetics of classical BZs and entered the market in the 1990s (143). Z-drugs are characterized by a rapid onset within 30 min and a short half-life from one to 7 hours (144). Compared to BZs that are approved for various condition, including anxiety, epilepsy, and insomnia, Z-drugs have only been approved for insomnia treatment.

Three medications belonging to the class of nonbenzodiazepine BZRAs were included in the Clinical Practice Guideline. All three drugs are FDA-approved for the treatment of insomnia. Zolpidem constitutes the most frequently described hypnotic medication in recent years worldwide (145). Zolpidem as well as eszopiclone were recommended for the treatment of sleep onset insomnia as well as sleep maintenance insomnia, while zaleplon, which has the shortest half-life of Z-drugs, was recommended for treatment of sleep onset insomnia only (95, 144) (**Table 1**).

#### 3.2.2 Melatonin and melatonin receptor agonists

Soporific effects associated with melatonin, a hormone secreted by the pineal gland, has been well established since the 1960s and clinical trials conducted in the 1970s implied sleep-promoting effects of melatonin (146). While melatonin exhibits both hypnotic and chronobiotic actions, it has some properties that limit its suitability as an oral agent for insomnia treatment, i.e. short half-life of (< 30 min) high first-pass metabolism, and binding to multiple melatonin receptors (147). With the characterization and cloning of the human melatonin receptors, MT1 and MT2 (148, 149), melatonin agonists were developed and entered the market in the mid 2000s.

Melatonin is marketed as a nutritional supplement. However, compared to other over-the-counter remedies, it has undergone more extensive evaluation regarding its effects as a hypnotic and chronobiotic agent (150). Melatonin as an over-the-counter treatment option was not recommended for sleep onset insomnia or sleep maintenance insomnia in the Clinical Practice Guideline (95) (**Table 2**). The recommendation was derived from studies in older adults (< 55 years) based on a lack of evidence for the efficacy of treatment compared to placebo and insufficient data concerning potential side effects.

**Table 2:** Drugs not recommended for treatment of chronic insomnia disorder.

Treatment	Dose	Recommendation	Strength	Ref.
Antidepressants (low-dose)				
Trazodone (FDA-approved, off-label use for insomnia)	50 mg	Use is **not** recommended for treatment of **sleep onset insomnia** or **sleep maintenance insomnia** (versus no treatment) in adults	WEAK	(124)
Anticonvulsants				
Tiagabine (FDA-approved, off-label use for insomnia)	4 mg	Use is **not** recommended for treatment of **sleep onset insomnia** or **sleep maintenance insomnia** (versus no treatment) in adults	WEAK	(151–153)
Over-the-counter medication				
Diphenhydramine	50 mg	Use is **not** recommended for treatment of **sleep onset insomnia** or **sleep maintenance insomnia** (versus no treatment) in adults	WEAK	(154)
Melatonin	2 mg	Use is **not** recommended for treatment of **sleep onset insomnia** or **sleep maintenance insomnia** (versus no treatment) in adults	WEAK	(155–157)
L-tryptophan	250 mg	Use is **not** recommended for treatment of **sleep onset insomnia** or **sleep maintenance insomnia** (versus no treatment) in adults	WEAK	(158)
Valerian and valerian-hops combination	variable	Use is **not** recommended for treatment of **sleep onset insomnia** or **sleep maintenance insomnia** (versus no treatment) in adults	WEAK	(154)

The table depicts dose, recommendation and its respective strength, and studies with sufficient quality for drugs not recommended for sleep onset insomnia and/or sleep maintenance insomnia in the Clinical Practice Guideline for the Pharmacologic Treatment of Chronic Insomnia in Adults (95). FDA, Food and Drug Association.Bold text indicates whether recommendations relate to sleep maintenance or sleep onset insomnia, respectively.

Ramelteon is a selective MT1 and MT2 receptor agonist with a considerably higher receptor affinity compared to melatonin. Ramelteon was approved by the FDA and indicated for the treatment of insomnia in 2005. It was recommended for the treatment of sleep onset insomnia but not for sleep maintenance insomnia in the Clinical Practice Guideline (95) (**Table 1**). The melatonin receptor agonists agomelatine that is approved for treatment of major depression in Europe, and tasimelteon that is FDA-approved and indicated for non-24-hour sleep-wake disorder, were not discussed as insomnia treatment options (95).

#### 3.2.3 Antidepressants

Prescriptions of antidepressants for insomnia treatment increased substantially over the 1980s and 1990s (159). By the beginning of the 2000s off-label prescriptions of antidepressants for insomnia treatment was about 1.5 times higher than prescriptions of hypnotic medications with FDA-approved indication for insomnia treatment (160). This steep increase that occurred albeit scientific evidence of efficacy was lacking at the time (161), might be attributed to the perception of clinicians that antidepressants constitute a safer option to BZRAs owing to the negative image regarding tolerance and withdrawal effects of the latter (161). Antidepressant drugs assessed in the Clinical Practice Guidelines include the tricyclic antidepressants (TCAs) doxepin and trimipramine, the serotonin antagonist trazodone, and the selective serotonin reuptake inhibitor (SSRI) paroxetine. Dosages of antidepressants for insomnia treatment are lower than those used for treatment of depression and can be considered sub-therapeutic for depression treatment.

Of the discussed antidepressants, only doxepin has been approved for the treatment of insomnia, while trazodone, paroxetine, and trimipramine constitute common off-label choices.

The TCA doxepin is highly selectivity for the histamine H1 receptor at very low doses. As histamine is considered one of the key neurotransmitters that mediate wakefulness (162), the hypnotic actions of doxepin can be attributed to its inhibitory effect on the H1 receptor (102). Very low-dose doxepin was the only antidepressant recommended for the treatment of sleep maintenance insomnia in the Clinical Practice Guideline (95) (**Table 1**).

Trazodone constitutes a serotonin antagonist and reuptake inhibitor. Its hypnotic effect at low doses has been attributed to the blocking of the 5-HT2A, histamine H1, and alpha receptors (163). While trazodone received FDA-approval for treatment of depression in 1982, the off-label use of the medication for insomnia treatment has since surpassed its prescription as an antidepressant in the US (164). Trazodone was not recommended for the treatment of sleep onset insomnia or for sleep maintenance insomnia in the Clinical Practice Guideline due to absence of clinically significant improvement regarding all assessed sleep outcomes (95) (**Table 2**).

Trimipramine as well as paroxetine were both reviewed in the Clinical Practice Guideline, but no recommendation regarding either drug was made due to insufficient evidence regarding safety and efficacy (95).

#### 3.2.4 Orexin receptor antagonists

The orexin system, comprising the two peptides orexin A and orexin B, and their target receptors orexin-1 and orexin-2, were first described by two independent working groups in 1998 (165, 166). The orexin system was originally described to be involved in feeding behavior and regulation of central energy expenditure, before first publications linked it to the sleep-wake cycle and established it as a potential target for treatment of sleep disorders (167–170). Similar to drugs that target the histamine H1 receptor, blocking of the orexin receptor system is based on the perspective of insomnia as a disorder of inappropriate wakefulness rather than a sleep disorder (171).

To date five dual and one selective orexin receptor antagonist (ORA) have been utilized in clinical practice or were included in clinical trials. Three of these compounds, i.e. suvorexant, lemborexant, and daridorexant, which all constitute dual ORAs, have been approved by the FDA and indicated for the treatment of chronic insomnia (172).

Only suvorexant that was the first dual ORA to receive approval for clinical use in 2014, was included in the Clinical Practice Guideline. Suvorexant was recommended for treatment of sleep maintenance insomnia but not for sleep onset insomnia (100). However, it is suggested that suvorexant might have beneficial effects on sleep latency at the higher dose of 20 mg (95).

Lemborexant and daridorexant entered the marked in the US after the Clinical Practice Guideline was published (Lemborexant in 2019 and daridorexant in 2022) (172). Additionally, daridorexant will constitute the first dual ORA to be approved for treatment of insomnia in the European Union. A systemic review and network meta-analyses published in 2022 by Xue and colleagues that assessed the efficacy and safety of dual ORAs in the treatment of primary insomnia found no significant differences between any of the ORAs with regard to subjective or objective parameters of sleep quality (173).

#### 3.2.5 Anticonvulsants

Two anticonvulsants were included in the Clinical Practice Guideline.

Tiagabine constitutes a selective GABA reuptake inhibitor that, by inhibiting the GAT-1 GABA transporter, increases synaptic availability of the neurotransmitter. Tiagabine is characterized by fast absorbance and has a half-life of 7 to 9 h (151). It has FDA-approval for adjunctive treatment of partial seizures, and has been proposed as an off-label treatment option for insomnia in older individuals (151). Tiagabine was not recommended for sleep onset insomnia or sleep maintenance insomnia in the Clinical Practice Guideline due to clinically insignificant effects on its efficacy (95) **Table 2**.

Gabapentin is a GABA analogue. It is not indicated for treatment of insomnia by the FDA and has been used off-label oftentimes in patients in whom other pharmacologic treatment options might be contraindicated (i.e. in the context of alcohol dependency) (174, 175). No recommendation was made regarding the use of gabapentin for insomnia treatment due to insufficient data (95).

#### 3.2.6 Antipsychotics

Quetiapine is the only antipsychotic included in the Clinical Practice Guideline. It belongs to the class of atypical antipsychotics and exhibits a high affinity for 5-HT2A receptor as well as weak affinities for dopamine, muscarinic, and adrenergic receptors. No recommendation for quetiapine was made due to insufficient evidence regarding efficacy and safety (95).

#### 3.2.7 Over-the-counter medication

Next to melatonin that was discussed in the according section above, non-prescription agents for the treatment of insomnia include sedating antihistamines as well as nutritional supplements and herbal remedies. Over-the-counter (OTC) sleeping aids are popular first-line treatment option for individuals with acute insomnia (176). However, studies suggest that especially older patients use OTC medication for longer than indicated (176). Albeit, the widespread use of OTC and nutritional supplements, including L-tryptophan and valerian, there are questions regarding their respective efficacy for insomnia treatment and their safety especially when used chronically (176).

Diphenhydramine (DPH) is an antihistamine with antimuscarinic properties. The sedative effect of DPH is brought forward by antagonism of the histamine H1 receptor. The Clinical Practice Guideline does not recommend use of diphenhydramine for treatment of sleep onset insomnia or sleep maintenance insomnia due to the absence of clear benefits (95) (**Table 2**).

The melatonin precursor tryptophan crosses the blood-brain barrier before being converted to serotonin and subsequently to melatonin. Similar to DPH no clinically significant effect on parameters of sleep quality or quantity was evident and therefore L-tryptophan was not recommended for treatment of sleep onset insomnia and sleep maintenance insomnia (95) (**Table 2**).

The sedating properties of valerian has been recognized since the 18^th^ century and its sedating effects have been attributed to inhibitory effects on the sympathetic nervous system via modification of the GABA system (177). While patients might prefer a natural remedy for treatment of insomnia, valerian and valerian-hops preparation in variable doses were not recommended for sleep onset insomnia and sleep maintenance insomnia due to the absence of demonstrated efficacy (95) (**Table 2**).

#### 3.2.8 Comparative effectiveness of pharmacological treatments of insomnia

A systematic review and network meta-analysis was published in 2022 that assessed to comparative effectiveness of pharmacologic treatment options of insomnia (178). Similarly, to the Clinical Practice Guideline, patients with physical comorbidities were not included in the respective analyses. Therefore, it remains unclear whether the results can be also attributed to patients with cardiovascular diseases. Nevertheless, this study provides a comprehensive overview regarding the comparative efficacy, tolerability, and safety of relevant pharmacologic treatment options for short- and long-term treatment of insomnia based on currently available literature, which is summarized in the following paragraph (178).

The respective meta-analysis included 154 studies that investigated the effect of 36 active pharmacological treatments or placebo in adults. The meta-analyses included a total 12,670 participants in the placebo groups and 35,280 participants in the respective treatment groups and effects of acute and long-term treatment were assessed. Clinical outcomes included parameters of efficacy, acceptability, tolerability, and safety. Importantly, attributable to the age of the included studies objective measures of sleep were often not available. Consequently, efficacy as primary outcome was evaluated based on subjective sleep quality. Overall, results suggested a favorable profile for the Z-drug eszopiclone that however might be associated with considerable adverse effects and for the dual ORA lemborexant albeit safety data were inconclusive. Several pharmacologic options were found to have positive data regarding tolerance, including doxepin, seltorexant, and zaleplon. However, respective results concerning efficacy were inconclusive due to a lack of data. While several prescription medications were found to be effective in short-term treatment, including BZs, daridorexant, suvorexant, and trazodone, these, however, were often associated with poor tolerability and data regarding long-term effects are missing. Similar as the Clinical Practice Guideline, melatonin, ramelteon, and over-the-counter medication overall failed to show beneficial effects in the treatment of insomnia. Overall, it needs to be considered that several limiting factors are highlighted by the authors. In this regard, only five of the included studies provided long-term data for more than four weeks and most comparisons were based on indirect evidence from a respective small subset of studies.

#### 3.2.9 Considerations regarding pharmacologic treatment of chronic insomnia in cardiac patients

Given the adverse outcome measures associated with insufficient sleep quality and duration, adequate treatment of chronic insomnia in patients with cardiovascular diseases is of importance (40, 45, 46). In this regard, it is noteworthy that the efficacy and safety of available treatment options for chronic insomnia have not been widely explored in patients with heart conditions and treatment recommendation for this clinical population are currently missing. The authors are unaware of according literature regarding pharmacologic interventions. Consequently, the *Clinical Practice Guideline for the Pharmacological Treatment of Chronic Insomnia in Adults* highlighted in this article are based on studies conducted with patients with primary chronic insomnia without any significant physical or mental comorbidities (95). Therefore, additional factors need to be considered when deciding on treatment options in cardiac patients. As recommended for the general population, before deciding on a specific treatment option for a patient, a comprehensive assessment of previous sleep complains, medical and psychiatric history, and information on medication and substance use should be performed (70, 95, 179). Additionally, the patient’s preferences as well as treatment availability should be taken into account. As in somatically healthy individuals, nonpharmacologic approaches, especially CBT-I, should be implemented as first line of treatment.

When deciding on pharmacologic therapies, which become clinically important in cases where CBT-I is unavailable, not tolerated by the patient, or ineffective, potential interactions with drugs utilized for the treatment of the cardiac condition have to be taken into account. Especially in patients with heart failure, treatment regimens are oftentimes complex and may lead to prolonged drug-elimination times. Consequently, particularly drugs with a longer half-life might be associated with an increased risk for daytime sleepiness.

Regarding pharmacologic options recommended by the Clinical Practice Guideline for treatment of sleep onset insomnia or sleep maintenance insomnia, little evidence regarding their efficacy and safety in cardiovascular patient populations exist. The following section summarizes limited data regarding pharmacologic treatment options and potential effects on the cardiovascular system and an according overview is given in **Table 3**. In this regard, while drug-drug interactions (DDI) between pharmacologic insomnia treatment approaches and cardiovascular medications are seldomly described, many psychopharmacologic drugs are metabolized via the cytochrome (CYP) P-450 pathway, and potential DDIs should be considered, i.e. by use of interaction databases.

**Table 3:** Relative risk of QTc prolongation associated with drugs frequently used for treatment of insomnia [adapted from (180)].

Treatment (substance class)	Interaction with CYP system	MOA	Side effect (main)	Side effect (cardiovascular)	Risk of QTc-prolongation
***Benzodiazepines***	-	Modulation of GABA_A_-receptor	Risk of abuse and addiction	Potential sedating properties on the respiratory system	–
***Z-drugs***	–	Modulation of GABA_A_-receptor	Risk of abuse and addiction	Potential sedating properties on the respiratory system	–
***Orexin-receptor antagonists***	CYP3A4	Blockade of Orexin-receptor 1 and 2 Suppression of wake-signals	Headache, nausea, tiredness	Potential beneficial effects on glycemic control and blood pressure	–
***Low potency antipsychotics***	Substance dependent, mostly CYP2D6	Dopamine receptor blockade (D_1_, D_2_, D_3_)	Sedation via blockade of H_1_- histaminergic receptors	Potential adverse metabolic side effects, weight gain, hypertension, T2D	Dose- and substance dependent increase in QTc
Melperone					+++
Chlorpromazine Levomepromazine Quetiapine					++
Promethazine, Pipamperone					+
***Antidepressants***	Substance dependent, mostly CYP2D6, CYP3A4, CYP2C19	Modulation of serotonergic and/or dopaminergic and/or noradrenergic neurotransmission	Substance dependent: sedation via blockade of H_1_-histaminergic receptors		Dose- and substance dependent increase in QTc
Tri- and tetracyclic antidepressants					++
Mirtazapine					(+)

CYP, cytochrome p-450; MOA, mode of action; T2D, type-2 diabetes mellitus. +++ high (> 20 ms); ++ moderate (10-20 ms); + low (< 10 ms); - no data or no risk described.Bold text indicates drug classes.

In a publication from 2008, while highlighting the lack of according data, treatment with ramelteon is suggested as a safe treatment for sleep disturbances in patients with systolic heart failure (181). This recommendation was based on the selectivity of ramelteon for the melatonin (MT)1 and MT2 receptor and the hypothesis that beta-blocker treatment that is common in this patient group, might be associated with impaired melatonin synthesis and secretion (182). While ramelteon is recommended for use in cases of sleep onset insomnia in the Clinical Practice Guideline, no recommendation was made for the treatment of sleep maintenance insomnia, due to clinically insignificant improvement in sleep quality and efficiency compared to placebo (95).

BZs have been attributed with sedating properties on the respiratory center. Therefore, their use for insomnia treatment in patients with cardiovascular disorders that often present with comorbidities regarding sleep-disordered breathing (183). This is substantiated by a recent study that compared the prognostic impact of BZs and Z-drugs in patients with heart failure and insomnia (184). Sato and colleagues report increased rehospitalization of patients that received a BZ for treatment of insomnia compared to those that received a Z-drug at discharge. However, it should be taken into account that patients were assigned to the respective study groups based on the prescription of hypnotic drug class at the time of discharge, and potential changes in medication or discontinuation were not assessed in the follow-up (184). Additionally, it might be considered that adverse impact on cognitive function that has been described for both, BZs and Z-drugs, particularly when prescribed long-term, might negatively influence adherence to cardiovascular drug treatment (185).

ORAs constitute relatively new pharmacologic options in the treatment of chronic insomnia and studies regarding their respective efficacy and safety in patients with cardiovascular diseases are currently limited. A multicenter study that included 82 patients with insomnia and treated hypertension assessed the effect of a two-week treatment with 20 mg/d suvorexant. While the study reported a significant improvement in self-reported total sleep time (TST) and time to sleep onset (TSO) as well as a significant decline in night time blood pressure, no significant differences compared to the placebo group were found (186). This is in contrast to previous studies with estazolam and zolpidem, respectively, that reported a significant decrease in blood pressure associated with a significant improvement in sleep quality (187, 188). Potential blood pressure lowering properties of orexin receptor antagonist are supported by pre-clinical studies in mice and rats that commonly reported hypertensive effects of orexin (189–191).

In a study that included 18 individuals with insomnia and type-2 diabetes, three-day treatment with suvorexant was associated with significant improved total sleep time and sleep efficacy, while change in sleep latency was not significant. Suvorexant treatment was associated with improvement in glycemic control (192). A recent study assessed the efficacy and safety of the dual ORA daridorexant in patients with mild to moderate obstructive sleep apnoea. The study that included 28 patients concluded that single, as well as repeated doses of 50 mg daridorexant did not negatively impact night time respiratory function and improved sleep parameters (193). Additionally, daridorexant was not associated with changes in cardiac repolarization in healthy volunteers (194).

Among the sedating antidepressant, only doxepin received a weak recommendation for the treatment of sleep maintenance insomnia at very-low dosage (95). Nevertheless, other sedating antidepressants are commonly prescribed as off-label treatment options as clinicians often perceive them as the safer pharmacologic treatment option compared to BZ and Z-drugs, especially in older individuals and as long-term treatment, i.e. 5 weeks or longer (195, 196). However, studies that assessed the safety and efficacy of these drugs in the context of depression and accordingly studies in the context of insomnia treatment are scarce and data regarding their efficacy as long-term treatment option are currently missing (195). As dosages for off-label insomnia treatment are commonly lower than those used for indicated treatments, reported cardiovascular side-effects might be less of a concern. However, clinicians should be aware of common cardiovascular and cardiometabolic side-effects attributed to antidepressants when deciding on insomnia treatments for patients with cardiovascular diseases. Potential adverse effects of antidepressants in the context of cardiovascular disease have been discussed in several dedicated review articles (180, 197, 198). Owing to the wide-spread use of antidepressants for insomnia treatment, a short overview regarding potential interactions of antidepressants with the cardiovascular system is provided in the following paragraph.

TCAs that are frequently used for insomnia treatment albeit in sub-therapeutic doses for treatment of depression (195, 199). Among TCAs, doxepin, trimipramine, and amitriptyline appear to be the most commonly prescribed for insomnia treatment (66, 195, 200). TCAs have been associated with strong cardiovascular side-effects attributable to their anticholinergic and chinidine-like properties. Described abnormalities in cardiac conduction in the context of TCA treatment include QTc prolongation, atrioventricular block, atrial fibrillation, and ventricular tachycardia (180, 197, 198). Additionally, a higher risk of stroke and myocardial infarction have been attributed to TCAs when compared to SSRIs (180, 197, 198).

Trazodone is a heterocyclic antidepressant that is used frequently as an anti-insomnia drug, especially in the US (101). Trazodone has been associated with comparable cardiovascular side-effects to TCAs, including the induction of atrial and ventricular arrythmias especially in individuals that present with pre-existing cardiovascular disease (201). As trazodone has been reported to introduce polymorphous ventricular tachycardia and QT prolongation in the context of simultaneous amiodarone treatment, trazodone should not be given in combination with QT interval-prolonging drugs (201).

Mirtazapine constitutes a heterocyclic antidepressant with specific serotonergic and noradrenergic properties. It has been attributed with a comparatively favorable profile regarding cardiovascular side-effects that might include weight gain and orthostatic hypotension (180).

Agomelatine is considered in insomnia treatment due to its melatonergic properties and no evidence regarding cardiovascular side-effects have been published to our knowledge (180).

SSRIs appear to disrupt sleep in the early phases of treatment (202). Additionally, although generally considered safe for patients with cardiovascular disease, SSRIs have been associated with the potential to increase QTc intervals, which necessitates regular ECG monitoring (180).

Antipsychotics are divided into high potency and low potency drugs. Low potency antipsychotics are characterized by a low affinity for the dopamine receptor, but due to their high potency for histaminergic receptors, exhibit sedative properties. Although not approved for insomnia treatment, low potency antipsychotics, including melperone, promethazine, pipamperone, prothendyle, chlorprothixene, and levomepromazine, are frequently prescribed. Low potency antipsychotics, including quetiapine, which constitutes the most commonly used antipsychotic for insomnia treatment, have been shown to be associated with a moderate risk for QTc prolongation (180). In general, low potency antipsychotics should be avoided, if no primary indication for its use is present, as these drugs can be associated with metabolic side-effects including weight gain, hypertension and type-2 diabetes (203). Furthermore, antipsychotics have been reported to be associated with impaired cardiac function, myocardial fibrosis and inflammation in patients with schizophrenia (204).

## 4 Conclusion

Insomnia is more frequent in patients with cardiovascular disease when compared to the general population. Although insomnia and short sleep duration have been shown to negatively impact outcome parameters in the context of cardiovascular disease, treatment guidelines regarding the treatment of chronic insomnia in this patient population are currently missing. Based on studies and guidelines for insomnia treatment in individuals without additional somatic diseases, non-pharmacologic treatment options, especially CBT-I, should also be first-line treatment choices in patients with cardiovascular diseases. Pharmacotherapy should only be considered when other options are not available or failed to improve the condition. Available pharmacologic treatment options are mostly licensed for short-term treatment up to four weeks. Importantly, the type of sleep complaint, i.e. sleep onset insomnia versus sleep maintenance insomnia, or both, needs to be considered when choosing a hypnotic drug for a patient. Among the drugs with indication for insomnia treatment, Z-drugs should be preferred over BZs for treatment of patients with cardiovascular disorders, based on their sedating effects on the respiratory center and on recent research indicating adverse outcomes among BZ-treated patients with heart failure. Additionally, BZs are often associated with safety concerns, due to their longer half-life and higher risk of dependence and habituation compared to Z-drugs. Off-label treatment of insomnia with sedating antidepressant is common, especially when long-term treatment is considered. However, evidence regarding efficacy is scarce. When considering antidepressant treatment for insomnia in cardiac patient populations, clinicians should be aware of the potential side-effects on the cardiovascular system and respective safety measures, i.e. ECG monitoring, should be considered. Finally, ORAs constitute a newer drug class for insomnia treatment that is licensed also for long-term treatment. However, studies regarding their safety in patients with cardiovascular disease are currently missing.

Overall, studies regarding insomnia treatment of patients with cardiovascular disease, regarding efficacy and safety of commonly prescribed drugs are missing. Given the high frequency of insomnia in this patient population and the adverse outcomes associated with insomnia, studies regarding treatment efficacy and safety of insomnia medications as well as clinical treatment guidelines concerning insomnia treatment in cardiac patients are urgently needed.
