# Supplementary material for: Pharmacological and psychological approaches to insomnia treatment in cardiac patients: a narrative literature review
Source: Front Psychiatry. 2025 Feb 13;16:1490585. doi: 10.3389/fpsyt.2025.1490585 (PMC11865029; doi:10.3389/fpsyt.2025.1490585)
Supplement: Supplementary file 1 [file DataSheet1.pdf]

frontiers

Supplementary Material

1 Supplementary Tables

Supplementary Table 1. Keywords utilized for literature search according to search element.

Insomnia	Cardiovascular Disease	Therapeutic Options
Insomnia	Cardiovascular disease	Pharmacologic treatment
Chronic insomnia	Cardiac disease	Antidepressant
Sleep disorder	Cardiometabolic disorder	SSRI
Sleep disturbances	Chronic heart failure	TCA
Sleep duration	Heart disease	Doxepin
Sleep latency	Heart failure	Trazodone
Sleep maintenance	Arrhythmia	Antipsychotic
Sleep quality	Atrial fibrillation	Quetiapine
Sleep-disordered breathing	Coronary artery disease	Benzodiazepine
	Coronary heart disease	Temazepam
	Hypertension	Triazolam
	Myocardial infarction	Z-drugs
		Eszopiclone
		Zaleplon
		Zolpidem
		Melatonin
		Ramelteon
		Orexin
		Daridorexant
		Lemborexant
		Suvorexant
		Psychologic treatment
		Cognitive behavioral therapy
		CBT-I
		Acceptance and commitment therapy
		ACT
		Mindfulness
		Treatment guideline
		Insomnia treatment
